# Rheological Study of the Formation of Pullulan Hydrogels and Their Use as Carvacrol-Loaded Nanoemulsion Delivery Systems

**DOI:** 10.3390/gels9080644

**Published:** 2023-08-09

**Authors:** Esther Santamaría, Leticia Anjinho de Barros, Carme González, Alicia Maestro

**Affiliations:** Chemical Engineering and Analytical Chemistry Department, Faculty of Chemistry, Universitat de Barcelona Marti i Franques, 1, 08028 Barcelona, Spain; lanjinde7@alumnes.ub.edu (L.A.d.B.); carme.gonzalez@ub.edu (C.G.); amaestro@ub.edu (A.M.)

**Keywords:** pullulan gel, rheology, carvacrol, kinetics release, gelation time, swelling degree, hydrogel rehydration

## Abstract

Hydrogels have been extensively studied as delivery systems for lipophilic compounds. Pullulan hydrogels were prepared, and their gelation kinetics were studied over time. Pullulan exhibited a relatively slow gelling reaction in basic medium (KOH) using trisodium metaphosphate (STMP) as a cross-linking agent, so capsules cannot be obtained by dripping as easily as in the case of alginate and chitosan. The kinetics of pullulan gelation were studied through rheological analysis over time. An optimal [Pullulan]/[KOH] ratio was found for a fixed [Pullulan]/[STMP] ratio. For this given relationship, gelling time measurements indicated that when the concentration of pullulan increased, the gelation time decreased from 60 min for 6% *w*/*w* pullulan to 10 min for 10% *w*/*w*. After the gel point, a hardening of the hydrogel was observed over the next 5 h. The formed hydrogels presented high degrees of swelling (up to 1800%). Freeze-dried gels were capable of being rehydrated, obtaining gels with rheological characteristics and visual appearance similar to fresh gels, which makes them ideal to be freeze-dried for storage and rehydrated when needed. The behavior of the hydrogels obtained as active ingredient release systems was studied. In this case, the chosen molecule was carvacrol (the main component of oregano oil). As carvacrol is hydrophobic, it was incorporated into the droplets of an oil-in-water nanoemulsion, and the nanoemulsion was incorporated into the hydrogel. The release of the oil was studied at different pHs. It was observed that as the pH increased (from pH 2 to pH 7), the released amount of carvacrol for the gel with pullulan 10% *w*/*w* reached 100%; for the other cases, the cumulative release amount was lower. It was attributed to two opposite phenomena in the porous structure of the hydrogel, where more porosity implied a faster release of carvacrol but also a higher degree of swelling that promoted a higher entry of water flow in the opposite direction. This flow of water prevented the active principle from spreading to the release medium.

## 1. Introduction

A hydrogel can be considered a semisolid fluid where a macromolecule extends in the dilution media and interacts with neighboring ones, forming a three-dimensional network that retains high amounts of water. Broadly, there are different gel-forming strategies characterized by their cross-linking nature, both physical and chemical. Physical cross-linking methods are known for their reversible nature. On the other hand, chemical cross-links generally produce more rigid structures characterized by mechanical properties typical of solid 3D networks [1]. The properties of physically cross-linked hydrogels depend on their molecular structure, their synthesis [2], and their cross-linking density. If the cross-linking density of a cross-linked hydrogel is very high, it becomes brittle and shows poor mechanical properties [3,4].

Chemically cross-linked hydrogels are achieved by different covalent or ionic bonds [5,6], such as tough chemically cross-linked double-network hydrogels [7,8,9]. In the case of the formulation of alginate-based hydrogels, where the gelation process occurs in the presence of divalent ions, usually CaCl_2_, physically cross-linked hydrogels are obtained. Recently, mechanical and rheological characterization of biopolymer hydrogels has attracted great attention since mechanical and rheological properties have been proven to be important design parameters to be considered for the engineering of hydrogels in different fields such as biomedical [10,11,12,13] and food industries [14]. Alginate-based hydrogels have been widely studied [1,15,16,17,18,19], as have chitosan gels [20,21,22,23,24,25], but some biopolymers, such as pullulan, have not been so studied. On the basis of their composition of cross-linked polymeric components, hydrogels may be homopolymeric or multipolymeric. Pullulan has been introduced as a copolymer in the formation of multipolymeric hydrogels by mixing it up with gellan [26], alginate [27], or chitosan [28], among others [26]. However, although pullulan has increased its use for the formulation of edible films, there are few studies that prepare and study pullulan homopolymeric hydrogels. 

Pullulan is a polysaccharide produced by different *Aureobasidium pullulans*. Pullulan is a linear and unbranched polymer with maltotriose repeating units connected by α-(1 → 6) glycosidic bonds [29]. The first study using pullulan as a unique polymeric compound for the obtention of hydrogels analyzed the influence of (NaOH)–pullulan and sodium trimetaphosphate (STMP)–pullulan ratios in the rheological parameters of the formed gels using pullulan concentrations in the range 20–30% *w*/*w* [30]. The same authors proposed a mechanism to describe the reactions involved during the hydrogel synthesis [31] based on the research of Mocanu et al. [32]. 

**Figure 1:**
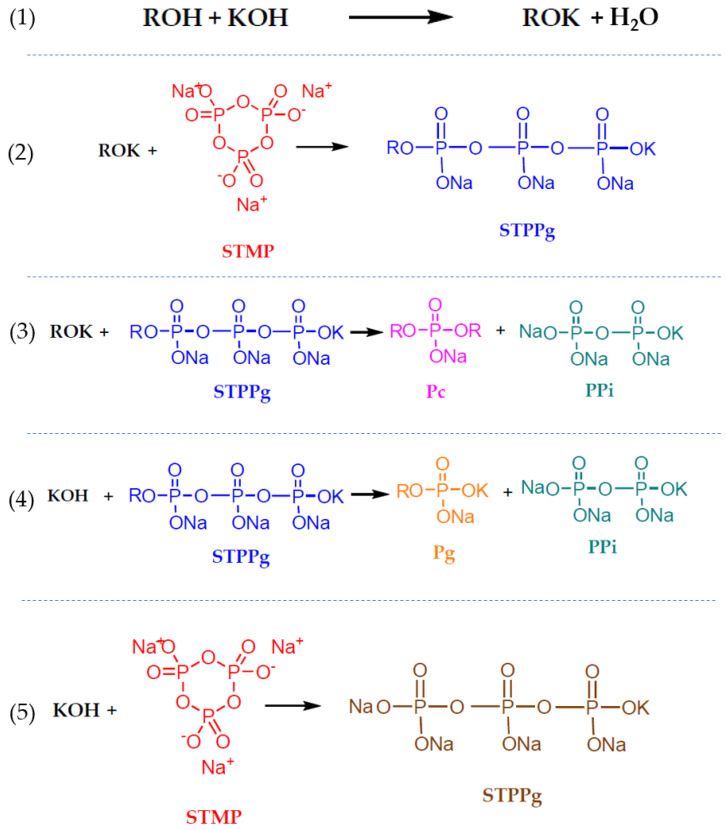
Proposed mechanism of the cross-linking reaction of pullulan with STMP [31].

The first step (Figure 1(1)) shows the reaction of pullulan in basic media with the hydroxyl group. The modified pullulan (ROK) can react with the STMP, becoming grafted to the sodium tripolyphosphate (STPPg) (Figure 1(2)). The STPPg-grafted pullulan is involved in the third reaction (Figure 1(3)) with another pullulan chain, which produces the cross-linked pullulan (Pc), the desired compound, and inorganic pyrophosphate (PPi). Meanwhile, two undesirable reactions may occur. Reaction (4) uses some of the STPPg to obtain grafted pullulan (Pg), avoiding the possibility of cross-linking more pullulan. Another undesirable reaction uses the reactants KOH and STMP to produce sodium tripolyphosphate (STPP), which is not useful for the pullulan cross-linked mechanism. The role of STMP as cross-linked agents was studied by rheological small-amplitude oscillatory shear measurements [2]. 

Some authors prepared pullulan gels for different uses [33,34,35], but these hydrogels have not been studied in depth from a rheological point of view. The reactions described in Figure 1 are relatively slow compared with the gelation mechanisms of other polymers such as alginate–CaCl_2_ [36], gellan–CaCl_2_ [37], carrageenan–CaCl_2_ [38], and chitosan–trisodium polyphosphate [39], among others [40,41,42]. The pullulan–STMP reaction kinetics do not allow the formation of gelled beads using the conventional dripping gelling method. Pullulan solution cannot be dripped over the gelling agent solution because this biopolymer disperses before gelation occurs, contrary to other biopolymers. However, hydrogels can be formed, and it is therefore relevant to study the gelling time as well as the properties of hydrogels depending on the composition variables. The ability of such hydrogels to act as carriers for active compounds can also be studied.

The formation of polymer gels can be monitored by measuring the viscoelastic properties of the material over time. The gel point is one of the most important physical parameters of the gelation process. At the gel point, a sudden loss of flow is observed due to the change of viscoelastic functions from an initial liquid-like state to a solid-like state. Consequently, the gel point can easily be determined from rheological data, as Winter’s method pointed out that at the onset of the gel point, the loss tangent, tan δ = G″/G′, becomes frequency independent [43].

The use of rheological measurements to determine the gel point of the hydrogels is well described. Atencio et al. [44] used a frequency sweep test to determine the gel point of alginate and alginate–kappa carrageenan hydrogels in function of the gelling agent concentration (CaCl_2_), and Ambebila et al. [37] studied gellan hydrogels. 

Hydrogels can be used as lipophilic active compound release matrices [45]. An efficient way to incorporate lipophilic compounds into hydrogel matrices is through the use of nanoemulsions. Direct oil-in-water (O/W) nanoemulsions can incorporate hydrophobic substances in the dispersed phase of the nanoemulsion [46,47,48,49]. This nanoemulsion is incorporated into the aqueous mixture of the hydrogel before its gelation, so that cross-linking of the molecules occurs in the continuous phase of the nanoemulsion and the hydrogel is formed with the oil droplets inside its matrix.

The use of essential oils is increasing due to their interesting benefits. Among the best-known oils are carvacrol, thymol, eugenol, linalool, cinnamaldehyde, D-limonene, etc. Each of these is mostly found in a different plant and has different characteristics and properties [45,46,47,48,49,50]. Carvacrol has been proven to be one of the essential oils with the strongest antimicrobial and antioxidant properties. Since this compound has a very low solubility in water, a way to add it to aqueous media is in the form of an oil-in-water emulsion (O/W) loaded into the hydrogel matrix. 

In this study, the formation of pullulan gels is monitored through the evolution of their rheological properties. This study presents the novelty of obtaining pullulan hydrogels with relatively low concentrations; those described so far used between 20 and 30% *w*/*w* of pullulan [31,32], while in this case we used percentages of pullulan between 610% *w*/*w*. These hydrogels are also obtained without the presence of another gelling agent, which allows for hydrogels with high transparency and high degrees of swelling.

Determining the gel point and obtaining hydrogels that gelate at different times broadens the range of possible applications of gels depending on the transition time from the liquid-like state to the solid-like state.

The influence of the composition of reactants on the sol-gel transition and gel strength, such as the pullulan–KOH and pullulan–STMP relationships, has been studied in order to find the optimal relationship. Some characteristics of the obtained gels were studied, such as the swelling degree, the water retention degree, the morphology of the freeze-dried gels, and their capacity to be reconstituted. The hydrogels were used as carvacrol-loaded nanoemulsion delivery systems in order to check their potential as shell materials for food applications. 

## 2. Results and Discussion

### 2.1. Gelation Kinetics

**Figure 2:**
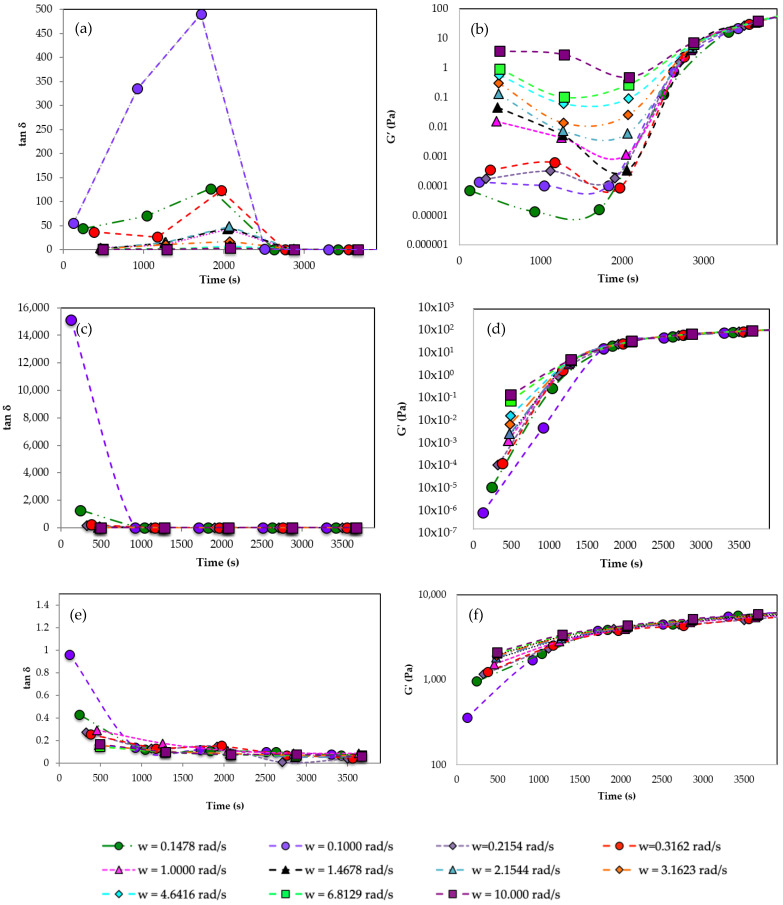
Tan δ at different frequencies vs. time. (**a**) Pullulan 6% *w*/*w*, (**c**) Pullulan 8% *w*/*w* (**e**) Pullulan 10% *w*/*w*. Storage modulus at different frequencies vs. time. (**b**) Pullulan 6% *w*/*w*, (**d**) Pullulan 8% *w*/*w* (**f**) Pullulan 10% *w*/*w*. All experiments set [pullulan]/[KOH] ratio of 3.5 and [pullulan]/[STMP] ratio of 2.

In order to evaluate the reaction time needed for the proper development of the gels, several studies changing the pullulan, STMP, and KOH concentrations were performed. In Figure 2, the loss tangent of the gel, tan δ, was plotted at several oscillation frequencies vs. time. According to Winter and Chambon [51], at the gel point, tan δ is independent of frequency, and, as a result, the gel point can be calculated as the point where all the curves collapse, as the gel point is strictly dependent on the material. The gel point is determined where loss tangent becomes independent of frequency and G′ becomes higher than G″ [43]. In Figure 2, gelation time for a [pullulan]/[KOH] ratio of 3.5 and a [pullulan]/[STMP] ratio of 2 is shown. Tan δ, and as a consequence, G′ values collapse for all the frequencies around 3600 s, indicating that in approximately one hour the gelation reaction has progressed far enough to form a well-structured gel extended to the whole bulk. As pullulan concentration increases from 6% *w*/*w* to 8% *w*/*w*, the gelation time decreases from 60 min to 30 min, and for a concentration of pullulan 10% *w*/*w*, the gelation time becomes shorter, reaching gel behavior in 10 min (Figure 2c,e). The higher the concentration of pullulan, the easier it is for pullulan molecules to cross-link with each other, forming a three-dimensional network. G′ is also shown in Figure 2b,d,f for different pullulan concentrations. The results showed that when the pullulan concentration increases, the gelation time becomes shorter.

**Figure 3:**
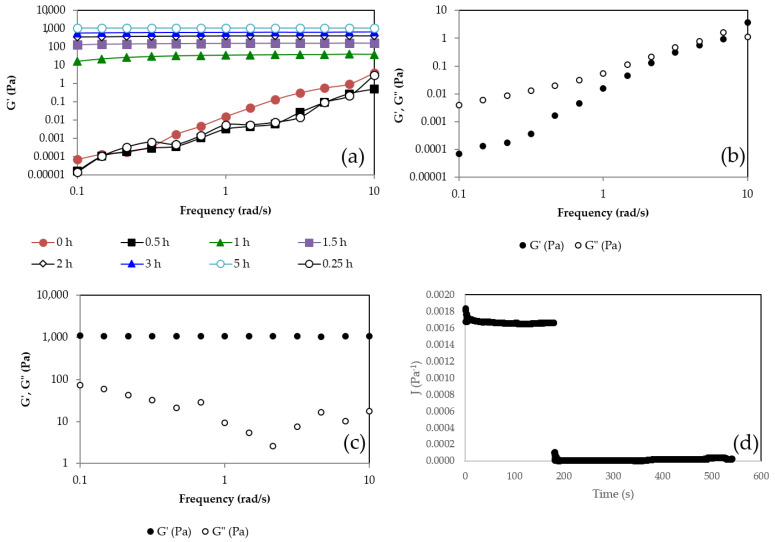
(**a**) Storage modulus (G′) vs. frequency at different times; (**b**) G′ and loss modulus, G″ vs. frequency at *t* = 0 h; (**c**) G′ and G″ vs. frequency at *t* = 5 h; (**d**) Creep-recovery test for τ = 3 Pa for *t* = 24 h. Pullulan 6% *w*/*w*, KOH 3% *w*/*w* and STMP 3% *w*/*w* at 24 h after the gel formation.

In order to evaluate the evolution of the gel once it was formed, its viscoelastic properties were measured over time. Frequency sweep tests were carried out. Figure 3a shows the storage modulus of the gel vs. frequency at different times. G′ at the initial time increases with the frequency, indicating that the mixture is not a gel and that its structure is evolving over time. At 1 h, it can be observed how the hydrogel has already formed because G′ is frequency independent. Above this time, G′ continues increasing with time due to the fact that the hardening reaction continues, forming a more compact network with a higher density of cross-links. At 5.5 h, the G′ value reaches its highest value, around 1100 Pa, which is 30 times greater than the value reached at the gel point (35 Pa). It can be observed that at time 0 h (Figure 3b), G′ and G″ increase with frequency, and G′ is lower than G″ in nearly all the range of frequencies, showing a liquid-like behavior. In addition, both moduli have small values. On the contrary, at a time of 5 h (Figure 3c), it can be seen that G′ is much higher than G″ and nearly independent of frequency. Above the gelation time, the storage component G′ is higher than G″ and tends to be independent of the frequency. 

The creep-recovery test (Figure 3d) is a mechanical test performed on materials to study their viscoelastic properties, measuring their instant and continuous deformation under a constant shear stress over time and their ability to recover their original shape once the shear stress is removed. The instant deformation is related to the elastic behavior and tends to disappear once the imposed shear stress is removed. The increasing deformation with time is related to flow and, therefore, to viscous behavior and is not recovered because the energy is dissipated due to friction under flow. In order to properly carry out the creep-recovery test, it is required to apply small shear stress to avoid breaking the structure and work in the linear viscoelastic range (LVR). Under these conditions, the compliance function, *J*(*t*) = deformation/applied shear stress, is independent of the imposed shear stress. A creep-recovery test was performed at *t* = 24 h after the formation of the gel in order to ensure that it was perfectly developed. Figure 3b shows that the compliance function suddenly increases at time zero when the shear stress is imposed and remains constant while the shear stress is applied, indicating an elastic deformation and the absence of flow. Once the shear stress is removed, at a time around 180 s, the compliance function decays to zero, indicating a complete recovery of shape, typical of perfectly elastic solids that follow the Hooke law, τ=G·γ, where τ is the imposed shear stress, *γ* is the deformation, and *G* = 1/*J* is the elastic modulus. Therefore, once the gel point is reached, a viscoelastic solid is formed that just increases its *G* value, hardening over time until a maximum *G* value is reached.

The [pullulan]/[STMP] ratio was set to 2, because preliminary experiments showed that for most of the experiments, a relatively hard and easy-to-demold gel was formed. Once this ratio was established, the influence of the amount of KOH used was evaluated. Table 1 shows the storage modulus, G′, for different pullulan concentrations and different [pullulan]/[KOH] ratios for a [pullulan]/[STMP] ratio equal to 2. Viscoelastic tests were performed 24 h after gel formation to ensure that all gels had evolved (Figure 2 showed that gels can evolve and harden within the first few hours) and to be able to compare their characteristics under the same conditions. So, all the hydrogels were fully developed. It can be observed that for the three pullulan concentrations evaluated, 6, 8, and 10% *w*/*w* at a [pullulan]/[KOH] ratio of 3.5, the value of the storage modulus reaches a maximum that is higher at higher [pullulan]. It can be observed that as the pullulan concentration increases at a low [pullulan]/[KOH] ratio, the gel does not form. As the pullulan increases, the minimum [pullulan]/[KOH] ratio for the formation of a gel moves to higher values.

The fact that gels are not formed when the [pullulan]/[KOH] ratio is low while the [Pullulan]/[STMP] ratio is kept constant indicates an excess of KOH that favors reactions 4 and 5 that the amount of KOH available per pullulan molecule in the reaction medium increases. As can be seen in Figure 1, the reaction mechanism for the gelation of pullulan includes two undesired reactions (reactions (4) and (5)) in which KOH intervenes. In reaction (4), the KOH reacts with STPPg, which is a necessary reagent for the formation of cross-linked pullulan, spending that reagent and preventing the pullulan molecules from cross-linking. In reaction (5), the KOH reacts with the STMP, opening the STMP chain as pullulan does but without the biopolymer chains, again using part of the reagent to obtain an unwanted product. Both reactions favor a high concentration of KOH.

**Figure 4:**
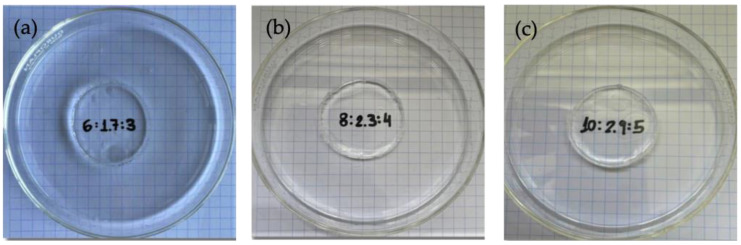
Gels formed at the optimum [Pullulan]/[KOH] ratio of 3.5, [Pullulan]/[STMP] ratio of 2 for (**a**) Pullulan 6% *w*/*w*, (**b**) pullulan 8% *w*/*w*, and (**c**) pullulan 10% *w*/*w*.

Figure 4 shows the images of the three gels obtained at the three pullulan concentrations and the best [pullulan]/[KOH] ratio in terms of storage modulus behavior. The hydrogels presented a compact structure, and their transparency degree was very high, as shown in Figure 4. The numbers written on the paper are perfectly legible across the petri dish with the gel, and no changes in color in the paper or clarity of the grid are observed. The hydrogels presented a very smooth touch, and they were easily manipulated.

### 2.2. Swelling Degree

**Figure 5:**
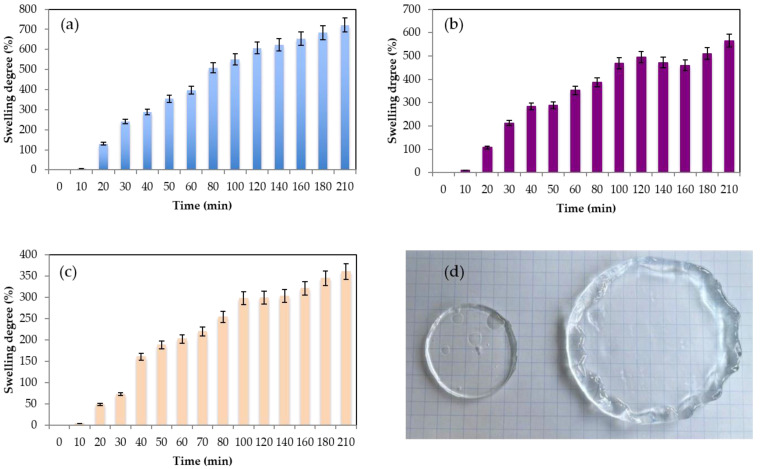
(**a**) Evolution swelling degree at different pullulan concentrations (**a**) 6% *w*/*w* (**b**) 8% *w*/*w* (**c**) 10% *w*/*w* and (**d**) image of the gel before and after swelling experiments for pullulan 6% *w*/*w*. For all experiments, the [pullulan]/[KOH] ratio was 3.5 and [pullulan]/[STMP] ratio was 2.

The swelling degree of hydrogels refers to the extent to which the formed hydrogel can absorb and retain extra water or other solvents when submerged in them. It is a measure of the hydrogel’s ability to swell or expand in the presence of a solvent. Swelling is possible since the solid component of the hydrogels, which is typically a polymer network, is both elastic and hydrophilic, so the water can be absorbed, increasing the hydrogel volume. The polymer creates elastic tensions within the cross-linked chains that prevent its total dissolution [52]. The swelling behavior of hydrogels is influenced by several factors, including the polymer composition, cross-linking density, pH, temperature, and nature of the solvent. The swelling behavior of hydrogels is important in various applications. For example, in agriculture, hydrogels can be employed to improve water retention in soil. Additionally, in material science, hydrogels with specific properties can be used in sensors, actuators, and smart properties. In biomedical applications, hydrogels with controlled swelling properties can be used for drug delivery, wound healing, tissue engineering, and as scaffolds for cell growth. The capacity of hydrogels to retain and absorb water is one of the crucial parameters to be evaluated in wound healing to assess the potential ability of the hydrogels to absorb excess wound exudates and hence maintain a suitable microenvironment in the wound [53,54]. Figure 5 shows the evolution of the swelling degree of the gels during the first 210 min. In Figure 5d, it can be visually observed how the pullulan gel strongly swells due to its high capacity for absorbing water. All the gels increased their weight, with the hydrogel obtained with 6% of pullulan being the one with the highest water absorbing capacity (Equation (1)) of 722% ± 56% at time = 210 min, followed by the hydrogel with 8% *w*/*w* with a swelling degree of 565% ± 37% and 360% ± 27% for the gel with 10% *w*/*w* of pullulan. After 24 h, the swelling degree was 1800% for the 6% *w*/*w* pullulan hydrogel, 1260% for the 8% *w*/*w* gel, and 990% for the 10% *w*/*w* gel. It seems that a less cross-linked network with less rigidity is better able to capture large amounts of water. Zawani et al. [53] obtained chitosan gels with very high swelling degree ratios of 1200% after 24 h, but when a denser cross-link was promoted with the addition of quercetin group, the swelling degree of hydrogels decreased until 350%, indicating that they had a lower capacity to catch extra water into their structure. Pullulan has proven to be a suitable biopolymer for wound healing, as Mert et al. [55] proved by obtaining pullulan hydrogels by UV copolymerization. Their hydrogels presented swelling degrees of 700% in neutral and basic media. Moreover, the gels obtained using STMP as the cross-linking agent in basic media presented better swelling degrees than the ones reported by Kalia et al. [56], where the addition of pullulan and xanthan biopolymers did not affect the gellan swelling degrees, around 120%, and they could not enhance the water absorption of the hydrogel. Tang et al. [21] reported the use of chitosan/(poly)vinyl alcohol hydrogels with poor swelling properties due to the compacted gel structure from which poly(vinyl) alcohol originated. As Sornkamnerd confirmed [57], the smaller pores and a subsequent stronger interlayer interaction between the biopolymer chains disturb the water molecules absorption into the hydrogels. The obtained hydrogels presented very good swelling behavior compared to the gels described before. It was observed that after 24 h of swelling of the gels, they presented a degree of water retention (WR) (Equation (2)) of 926% ± 35% for the hydrogels obtained with 6% pullulan, 540% ± 20% for those obtained with 8% pullulan, And 415% ± 15% for those with 10% pullulan. After 24 h in the desiccator, the gels have lost approximately half of the water that they managed to absorb in the previous 24 h. The hydrogels presented good results compared with the ones obtained with other kinds of biopolymers, such as chitosan [56], for which the WR was approximately 250% or 90% for hydrogels of pullulan with gellan [26].

### 2.3. Rehydration Behavior

**Figure 6:**
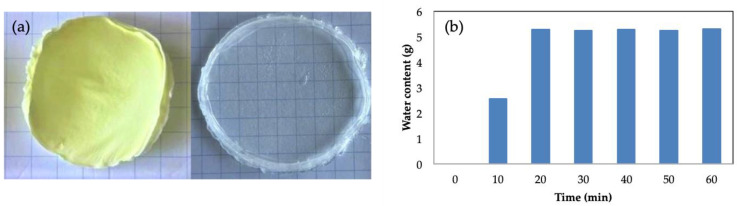
(**a**) Freeze-dried hydrogel and rehydrated gel; (**b**) evolution of the water content with time during the rehydration process for a pullulan concentration of 6% *w*/*w* and [Pullulan]/[KOH] ratio of 3.5, [Pullulan]/[STMP] ratio of 2.

Due to the great variety of uses that pullulan gels can have, it is interesting to evaluate the possibility of them being stored for later uses. One option is to subject them to a freeze-drying process. Different tests have been carried out in order to evaluate the properties of the gels after being subjected to this process. A photograph of a freeze-dried pullulan gel is shown in Figure 6a. It can be observed how the gel maintains its shape without breaking. The reconstitution process was carried out by placing the dry gel in the mold where the gels are formed and adding the same amount of water as in the fresh gels to ensure adequate swelling and reconstitution of the hydrogel structure. The final weight of the rehydrated gel was the same as the fresh gel. In Figure 6a, the image of the reconstituted gel is shown. It can be seen how the appearance of the gel was very similar to the gels obtained initially, with high transparency and a very similar size and shape. In Figure 6b, the water retention capacity of the freeze-dried gel is plotted vs. time of hydration. The weight of the dry gel was 0.25 g, and it is capable of absorbing up to 5 g of water. It can be seen how the gel absorbed water during the first 30 min and then no longer had the capacity to retain water. Considering that the weight of the fresh gel was 2 g, the freeze-dried gel had a degree of swelling of 150%. Valot et al. [58] studied the swelling behavior of their collagen freeze-dried hydrogels, obtaining a re-uptaking water degree of around 76–78%. The pullulan freeze-dried hydrogels obtained in this work presented a swelling degree of 150%, making the water absorption higher than that previously reported by Valot et al. [58]. Sornkamnerd et al. [57] evaluated the freeze-dried samples, showing a lower degree of water swelling than the fresh hydrogels. This was attributed to the contraction of the hydrogel structure during the drying process. The water retained in the hydrogel structure was removed, and the pullulan chains became closer together, reducing the porosity of the matrix. As previously discussed, the porosity of the hydrogels plays an important role in terms of water absorption. 

**Figure 7:**
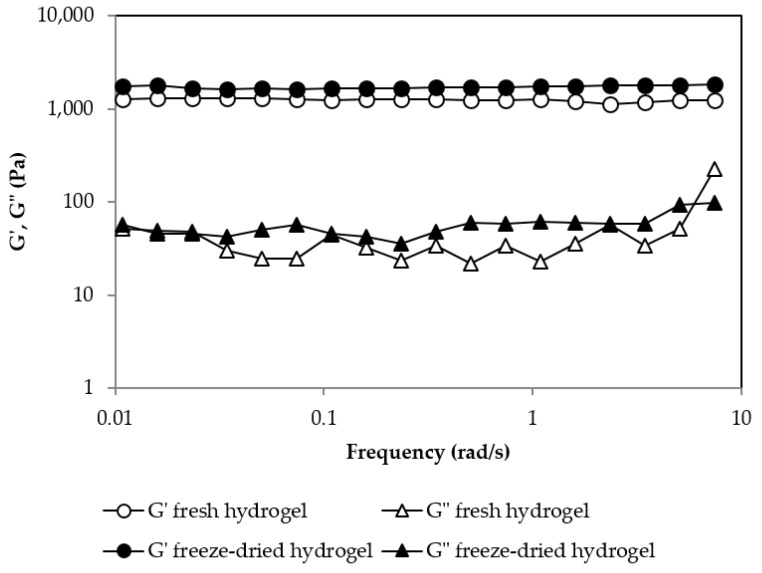
Oscillatory frequency sweep test for the fresh hydrogel and the freeze-dried hydrogel for a pullulan concentration of 6% *w*/*w* and [Pullulan]/[KOH] ratio of 3.5, [Pullulan]/[STMP] ratio of 2.

Figure 7 shows the rheological behavior of the gel before and after the reconstitution of the freeze-dried gel. It can be seen that in both cases the gel presented a gel-like behavior with a G′ frequency independent of and higher than G″. The G′ value for both gels was quite similar, indicating that the rehydration process was successfully completed and that it provided a gel with very similar mechanical properties to the fresh one.

### 2.4. SEM Characterization

**Figure 8:**
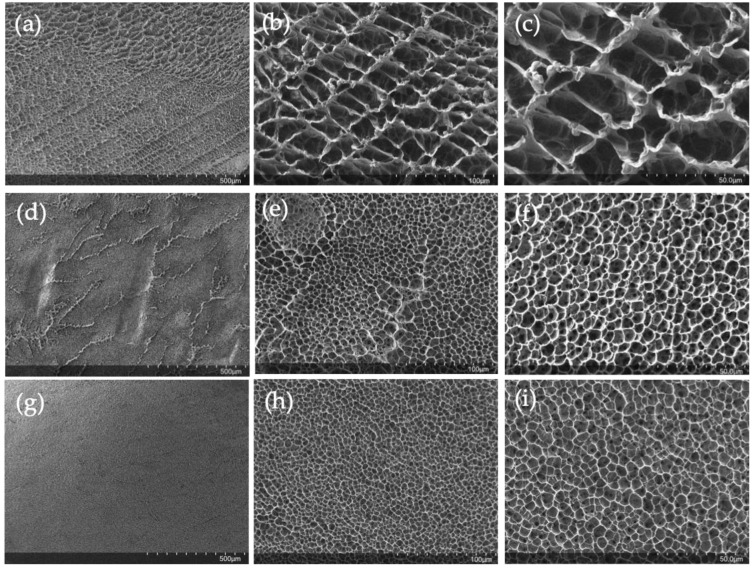
Freeze-dried hydrogel images at different magnifications for (**a**–**c**) pullulan 6% *w*/*w* (**d**–**f**) pullulan 8% *w*/*w* and (**g**–**i**) for pullulan 10% *w*/*w*.

The freeze-dried hydrogels were characterized by SEM microscopy. It can be seen how the pullulan network becomes more compact as the concentration of the gels increases (Figure 8a,d,f). For 6% pullulan (Figure 8a) at low magnifications, the pores are appreciated, while for a pullulan concentration of 10% *w*/*w*, the pores are not so easily distinguished (Figure 8g).

At higher magnifications (Figure 8b,e,h) the porous structure of the hydrogels is appreciated in more detail. The porous network becomes more compact with increasing pullulan concentration, and in the cases of 6% *w*/*w* pullulan (Figure 8b) and 10% *w*/*w* (Figure 8h), the pore network looks very uniform. The uniformity is also evident at the highest magnifications (Figure 8c,f,i). Once the pullulan gels have been characterized, it can be confirmed that the increase in the degree of swelling can be attributed to the structure of the hydrogel. As some authors reported [58,59], the degree of swelling depends on the hydrogel’s structure. As the network became more compact, the hydrogels had less ability to absorb and retain water in their structure. 

### 2.5. Carvacrol Release Kinetics

In order to determine the suitability of the hydrogels for delayed release of carvacrol under different pH conditions, the in vitro behavior in buffer solutions at 25 °C was studied. A carvacrol nanoemulsion was added as described in Section 4.3 in order to study the carvacrol release kinetics from the hydrogel to the medium. It was visually seen that the addition of the nanoemulsion did not affect the gel point since, once reached, the sample did not flow when the container was overturned. The pullulan–STMP cross-linked reaction was not affected by the addition of the dispersed phase. This was due to the fact that the nanoemulsions have very small droplet sizes, and the final amount of dispersed phase was low, being 2.50% *w*/*w* of total oil and 0.75% *w*/*w* of carvacrol.

**Figure 9:**
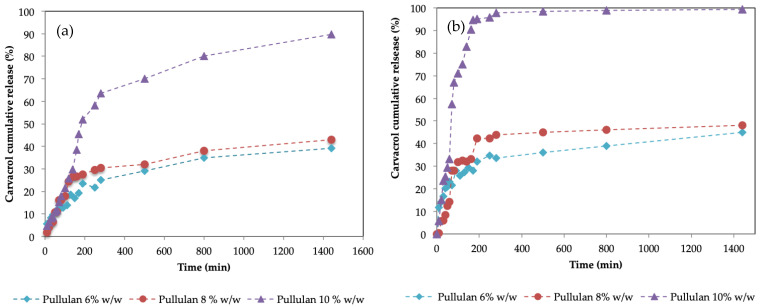
Cumulative carvacrol release of hydrogels synthesized using pullulan 6% *w*/*w* (blue points) pullulan 8% *w*/*w* (red points) and pullulan 10% *w*/*w* (purple points) for (**a**) pH = 2 and (**b**) pH = 7.

Figure 9 shows the cumulative carvacrol release patterns for hydrogels synthesized using 6%, 8%, and 10% *w*/*w*. The drug release study was carried out in duplicate in order to evaluate the reproducibility of the patterns. Surprisingly, although less porous, for both pHs, the hydrogels obtained with a higher pullulan concentration presented a faster release.

In Figure 9, it can be observed that for pullulan concentrations of 6% *w*/*w* and 8% *w*/*w*, the release of carvacrol is quite similar, although slightly higher for 8% and much less than for the case of 10% *w*/*w*. This occurs for both pHs. By increasing the pH from 2 to 7, the release of carvacrol increases, as other authors have previously reported [59,60,61,62]. SEM images of the hydrogels showed an increase in pore size with decreasing pullulan concentrations. It was expected that the larger the porous structure of the hydrogel, the easier it was for the active principle to leave the structure in which it was retained, and therefore it should present a greater release into the environment. This behavior has been described by some authors [63,64], especially in the case of porous materials, but also for hydrogels [65,66]. Experimental data show that in this case, this does not occur. This is presumably due to the high degree of swelling that hydrogels present, which is much higher for 6% pullulan. As previously described, these hydrogels have a high degree of swelling: 720% in the case of 6% *w*/*w*, 550% for 8% *w*/*w*, and 350% for 10% *w*/*w* at 210 min. This behavior was also observed by Wan et al. [67], who reported an inverse relationship between the release of the drug and the degree of swelling of the matrix, so that the degree of swelling is one of the factors that affect the release of active compounds, as corroborated by Carbinatto et al. [68]. Our results are consistent with those of Colombo et al. [69], who observed that the rate of delivery is dependent on the diffusion front velocity. They concluded that in fact, when the swelling front accelerated, indicating faster water penetration, but the diffusion front rate remained unchanged, the release kinetics were affected due to the water flow in the opposite direction to the flow of the active compound.

When the hydrogel swells, there is a flow of water that is directly entering the hydrogel structure, filling and enlarging the pores. This flow goes in the opposite direction to the release of carvacrol and slows down the releasing flow, leading to a competition between the incoming water and the carvacrol that diffuses into the medium.

Moreover, the incoming water reduces the driving force of the molecular diffusion by reducing the concentration of carvacrol inside the hydrogel due to dilution, as the driving force is (C_h_−C_0_), where C_h_ is the concentration of carvacrol inside the hydrogel and C_0_ is the concentration of carvacrol in the external medium [69].

According to Siepmann [70], the apparent diffusivity of the active component can be calculated using Equation (4). Since it is an equation that derives from the Higuchi model (Equation (3)), Table 2 shows the adjustment parameters of both. It can be observed how the Higuchi model presents relatively good fitting parameters and apparent diffusivity coefficients. The apparent diffusion coefficients presented higher values than the ones calculated by other authors [71,72], which obtained the effective carvacrol diffusivities in different kinds of matrix, commonly films, being around 10^−14^–10^−17^ m^2^/s depending on the temperature and the film. As far as the authors know, the diffusion coefficient of carvacrol from hydrogels has not been previously reported.

The apparent diffusivity coefficient increases with increasing % of pullulan in the hydrogels. For the values of 6% *w*/*w* and 8% *w*/*w*, the values are similar, while for the case of 10% *w*/*w*, this apparent diffusion is an order of magnitude greater. This fact reaffirms the idea that although with a high % of carvacrol, the porosity is lower and carvacrol would have to follow more tortuous paths than for the other concentrations in order to be released, it happens faster than for lower concentrations with bigger pores. As discussed above, a lower inlet water flow, due to the lower swelling behavior, would explain the greater rate of release observed at 10%.

## 3. Conclusions

In this work, pullulan hydrogels have been synthesized and their gelation kinetics over time have been studied. Pullulan exhibits a relatively slow gelation reaction in a basic medium (KOH) using sodium trimetaphosphate (STMP) as a cross-linking agent, compared to the gelation of alginate with calcium chloride or chitosan with STMP. Therefore, obtaining gel capsules is much more complicated. However, bulk hydrogels can be prepared and studied. It has been studied how the [pullulan]/[KOH] ratio affects the hydrogels obtained. It has been observed that there is an optimum in this relationship in reference to a maximum of the storage modulus of the gels obtained, and the ratio where the optimum is obtained does not change for the different concentrations of pullulan tested. The gelation time for this optimum, studied by means of rheological characterization, was observed to decrease from 60 min for concentrations of 6% *w*/*w* pullulan to 30 min for 8% pullulan and 10 min for 10% *w*/*w* pullulan. After this time, in which the mixture reticulates, forming the hydrogel, it was observed that this hydrogel evolved during the first 5–6 h, hardening until it reached a constant storage modulus value.

SEM characterization showed that the higher the pullulan concentration, the denser the hydrogel structure and the less porosity it presented. The hydrogels were freeze-dried and rehydrated to assess the storability of these hydrogels and their suitability to be reconstituted for subsequent applications. It was observed that the gel can be easily reconstituted, and its rheological and visual properties remain the same as those of the freshly prepared hydrogel.

The hydrogels presented high swelling degrees at a relatively short time (210 min), capturing 750% of water (weight of the swollen hydrogel/initial weight of the hydrogel) for pullulan hydrogels synthesized with pullulan 6% *w*/*w*, 350% for pullulan 8% *w*/*w*, and 350% for pullulan at 10% *w*/*w*. Swelling tests after 24h showed swelling degrees of 1800% for pullulan 6% *w*/*w*, 1260% for pullulan 8% *w*/*w*, and 990% for pullulan 10% *w*/*w*. It has been proven that the release of carvacrol is not proportional to the porosity of the hydrogels, and it is attributed to the higher incoming flow of water observed for gels with higher porosity, which slows down the diffusion of carvacrol towards the medium.

## 4. Materials and Methods

### 4.1. Materials

Food-grade carvacrol (W224502) with a purity ≥98%, potassium hydroxide pellets at 85%, sodium trimetaphosphate (STMP), and synthetic non-ionic surfactant Tween 80^®^ (P1754) were purchased from Sigma Aldrich Spain. Pullulan with a low M_W_ 4.2 × 10^5^ from ITW reagents was used. Oleic acid pharma grade (142,659.1611) with a purity of 88% was purchased from Panreac Spain. Medium-chain triglycerides (MCT) oil (Miglyol 812) was purchased from Acofarma Spain. The manufacturer reported a composition of 58.1% caprylic acid (C8:0) and 41.9% capric acid (C10:0). Water is deionized and further purified by milli-Q filtration.

### 4.2. Formation of the Gels

First of all, the necessary KOH for each sample was weighted in a tube, and the % of water was added. The tube was mixed in a vibromixer until all the KOH was dissolved. Then, the desired pullulan % w/w was weighted and mixed again until all pullulan was dissolved. The resulting mixture had a transparent appearance. Finally, the STMP was added and mixed for 1 min until a clear solution was obtained. The final amount of the mixture was 10 g. Several aliquots of 2.5 mL were immediately put into 35 mm diameter cylindrical molds. The volume used is necessary in order to fill all the molds with a height of 1 mm and obtain gels with an adequate shape to be measured with the selected geometry in the rheometer. 

### 4.3. Carvacrol Nanoemulsion Loaded into Pullulan Hydrogels

Carvacrol-loaded nanoemulsions were formulated as previously described by Santamaria et al. [73]. The final content of the nanoemulsions was 1.5% *w*/*w* carvacrol, 3.5% *w*/*w* miglyol 812, 3% *w*/*w* oleic acid, 7% *w*/*w* Tween 80^®^, and 85% *w*/*w* water.

Nanoemulsion-loaded hydrogels were formulated using the double concentration of the desired one, i.e., for the experiments with 6% *w*/*w* pullulan, 12% *w*/*w* was weighed as described in Section 4.2. Once the solution was obtained, 5 g of the pullulan solution was mixed with 5 g of the nanoemulsion, leading to a final mixed composition of 6% *w*/*w* of pullulan and 0.75% *w*/*w* of carvacrol in the final hydrogel. 

### 4.4. Rheological Measurements

The rheological characterization of pullulan hydrogels was carried out as a function of pullulan, STMP, and KOH concentrations. Oscillatory shear stress sweep preliminary tests were developed in order to determine the linear viscoelastic region (LVR). Then, frequency sweep tests were conducted with an oscillatory stress of amplitude 1 Pa, which was under LVR conditions. A HAAKE-MARS III rheometer (ThermoElectron GmbH, Karlsruhe, Germany) was used with a serrated parallel plate measuring geometry (35 mm diameter, 1 mm gap) to avoid slippage. The temperature was controlled at 25.0 ± 0.1 °C. The instrument allowed data processing using HAAKE RheoWin Data Manager software Version 3.12 (ThermoElectron GmbH). After loading, a resting time of 5 min was used before measurement to allow stress and temperature equilibration. Creep-recovery tests were developed with the same rheometer and geometry, applying a constant shear stress for 180 s and suddenly removing it while continuing the measurement of the deformation obtained. 

Frequency sweep tests were carried out for the determination of the gel point. The just-prepared liquid mixture was loaded into the rheometer, which was provided with a cap that had a small hole for the axis of the sensor. Various moistened gauze pads were placed inside the protective cap to prevent sample evaporation. The rheometer was programmed to carry out oscillatory frequency sweep tests at certain times in order to be able to scan the evolution of gel formation.

### 4.5. Swelling Degree and Water Retention

The amount of water-holding capacity (known as swelling degree (*SD*)) of the pullulan hydrogels was measured using the method described by Kalia and Choudhury [55]. First, the weight of the gel formed at 24 h (fresh gel) was recorded (*W_d_*). The gels were then submerged using a sieve in water in order to prevent eventual breaks, and the weight of the swollen gels was measured with time (*W_s_*).

(1) $$SD\;\left(\%\right)=\frac{\left(W_{s}-W_{d}\right)}{W_{s}}\cdot 100$$

Also, the water retention (*WR*) percentages of the hydrogels were evaluated. The swollen gels were placed in a desiccator for 24 h and weighted.

(2) $$WR\;\left(\%\right)=\frac{W_{s}}{W_{d}}\cdot 100$$

### 4.6. SEM Measurements

The samples were freeze-dried using a Christ lyophilizer equipment model Alpha 2-4 LD Plus for 24 h, and afterwards, the surface morphology of the gels was observed using a TM 4000 Plus Hitachi SEM microscope. Characterization was performed in the Nanostructured Liquid Characterization Unit at the Institute of Advanced Chemistry of Catalonia (IQAC), which belongs to the Spanish National Research Council (CSIC) and is affiliated with the NANBIOSIS ICTS of the Biomedical Networking Center (CIBER-BBN).

### 4.7. Carvacrol Release Kinetics Experiments

The release kinetics of the carvacrol nanoemulsions loaded into different pullulan gels were analyzed. A pullulan gel with a cylinder diameter of 35 mm and a height of 1 mm was put in 400 mL of buffer solution (pH: 2 and 7). The system was stabilized at 25 °C with mild magnetic stirring (50 rpm) in order to prevent gel breakdown. A total of 2 mL of the supernatant was extracted regularly and analyzed by spectrophotometry (Spectrophotometer UV-Vis 6305 Jenway). Subsequently, 2 mL of the solutions with the same pH were added to the corresponding system to maintain the same volume of liquid. The carvacrol concentration was detected at 276 nm [61]. The diffusion kinetics of entrapped carvacrol (0.75% *w*/*w*) across the hydrogel towards the aqueous solution were explored in relation to the microstructural properties of the matrix. The Higuchi model was used to fit the experimental data to Equation (3):

(3) MtM∞=kH·t12

where *M_t_* is the release at time *t*, *M_∞_* is the release at the end of the experiment, and *k_H_* is the Higuchi constant.

For each pullulan concentration, the apparent diffusion coefficient (*D_s_*) can be obtained from Equation (4) [70].

(4) MtM∞=4·Ds·tπ·h212

where *h* is the height of the disk/cylinder.

## Figures and Tables

**Table 1:** Storage modulus (G′) at different pullulan concentrations (%*w*/*w*) and [pullulan]/[KOH] ratio at a fixed [pullulan]/[STMP] ratio = 2.

[Pullulan] (%*w*/*w*)	[KOH] (%*w*/*w*)	[Pullulan]/[KOH]	[Pullulan]/[STMP]	G′ (Pa) at 24 h	Gel’s Appareance
6	5.0	1.20	2	514	Transparent slightly yellow
	4.5	1.33	2	630	Transparent slightly yellow
	4.0	1.50	2	348	Transparent slightly yellow
	3.5	1.70	2	1532	Transparent slightly yellow
	3.0	2.00	2	1243	Transparent slightly yellow
	2.5	2.40	2	1370	Transparent
	2.0	3.00	2	1276	Transparent
	1.7	3.50	2	1873	Transparent
	1.5	4.00	2	826	Transparent
8	5.0	1.20	2	*	Not gel
	4.5	1.33	2	76	Transparent slightly yellow
	4.0	1.50	2	426	Transparent slightly yellow
	3.5	1.70	2	385	Transparent slightly yellow
	3.0	2.00	2	752	Transparent
	2.5	2.40	2	2120	Transparent
	2.0	3.00	2	1856	Transparent
	1.7	3.50	2	2300	Transparent
	1.5	4.00	2	2170	Transparent
10	5.0	1.20	2	*	Not gel
	4.5	1.33	2	*	Not gel
	4.0	1.50	2	298	Transparent
	3.5	1.70	2	527	Transparent
	3.0	2.00	2	2059	Transparent
	2.5	2.40	2	2222	Transparent
	2.0	3.00	2	2243	Transparent
	1.7	3.50	2	5521	Transparent
	1.5	4.00	2	3465	Transparent

* Gel is not cross-linked after 24 h. Colors indicate optimal concentration for each gel.

**Table 2:** Parameters of the carvacrol release from different hydrogels at pH 2 and pH 7. K_H_ corresponds to Higuchi constant, R^2^ is the fitting goodness, and Ds is the apparent diffussion coefficient.

pH	[Pullulan] (%*w*/*w*)	Ratio [Pullulan]/[KOH]	Ratio [Pulllan]/[STMP]	K_H_	R^2^	Ds (m^2^/s)
2	6	3.5	2	0.0147	0.9556	7.74 × 10^−13^
	8	3.5	2	0.0239	0.9478	9.38 × 10^−13^
	10	3.5	2	0.0565	0.9690	2.12 × 10^−12^
7	6	3.5	2	0.0956	0.9937	2.37 × 10^−12^
	8	3.5	2	0.0348	0.9135	1.92 × 10^−12^
	10	3.5	2	0.0552	0.9587	1.07 × 10^−11^

## Data Availability

Data are contained within the article. Extra data will be provided on request.
